# Fetal Tachycardia Treated Successfully with Maternally Administered Propylthiouracil

**DOI:** 10.1155/2014/968051

**Published:** 2014-06-11

**Authors:** Barbara V. Parilla, Farhan Hanif, Keren Hasbani, Thomas Iannucci

**Affiliations:** ^1^The Division of Maternal-Fetal Medicine, Advocate Lutheran General Hospital, Park Ridge, IL 60068, USA; ^2^Parkside Center, 1875 W. Dempster Street, Suite 325, Park Ridge, IL 60068, USA; ^3^The Division of Pediatric Cardiology, Advocate Lutheran General Children's Hospital, Park Ridge, IL 60068, USA

## Abstract

*Background*. Fetal tachycardia may result from the transplacental passage of thyroid stimulating immunoglobulins in a patient with hypothyroidism secondary to ablation of Graves' disease. *Case*. A 32-year-old woman, gravida 4, para 2, and abortus 1, with hypothyroidism and a history of Graves' disease, presented at 23 6/7 weeks of gestation with a persistent fetal tachycardia. The treatment of the fetal tachycardia with maternally administered digoxin and Sotalol was unsuccessful. Maternal thyroid stimulating immunoglobulins were elevated, and treatment with maternally administered propylthiouracil (PTU) resulted in a normal sinus rhythm for the remainder of the pregnancy. An induction of labor was performed at 37 weeks. Four to five days after delivery, the neonate exhibited clinical signs of hyperthyroidism necessitating treatment. *Conclusion*. Fetal tachycardia resulting from the transplacental passage of thyroid stimulating immunoglobulins can be successfully treated with maternally administered PTU. The neonate needs to be followed up closely as clinical signs of hyperthyroidism may occur as thyroid stimulating immunoglobulins continue to circulate in the neonate, while the serum levels of PTU decline.

## 1. Introduction

Hypothyroidism is one of the most common disorders that affect adult women. Overt hypothyroidism occurs in 2% of female adults, and mild hypothyroidism affects approximately 2% of pregnant women and 5–17% of women older than 40 years. The most common cause of primary hypothyroidism is autoimmune thyroiditis, which increases in prevalence with age. Hypothyroidism also occurs frequently after radioiodine therapy and after surgery for hyperthyroidism, goiter, or thyroid cancer. The fetal risk of hyperthyroidism in women with a history of Graves' disease is not always appreciated, particularly in those women receiving thyroid replacement secondary to ablation or surgery. They may still be producing high levels of thyroid stimulating immunoglobulins which are able to cross the placenta and cause hyperthyroidism in the fetus [1–3]. We describe a case of fetal tachycardia secondary to the transplacental passage of thyroid stimulating antibodies, successfully treated with maternally administered PTU.

## 2. Case

**Figure 1:**
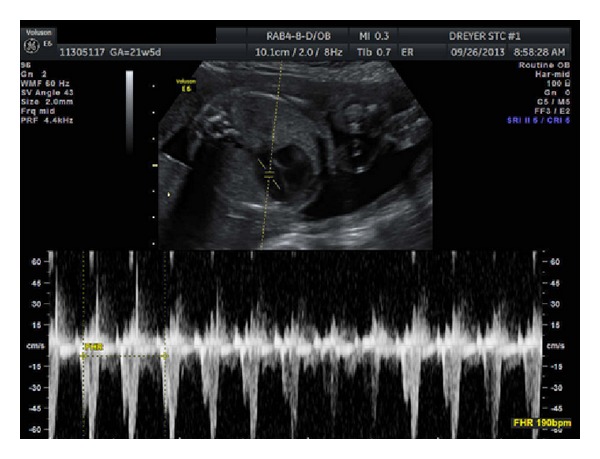
Fetal tachycardia.

**Figure 2:**
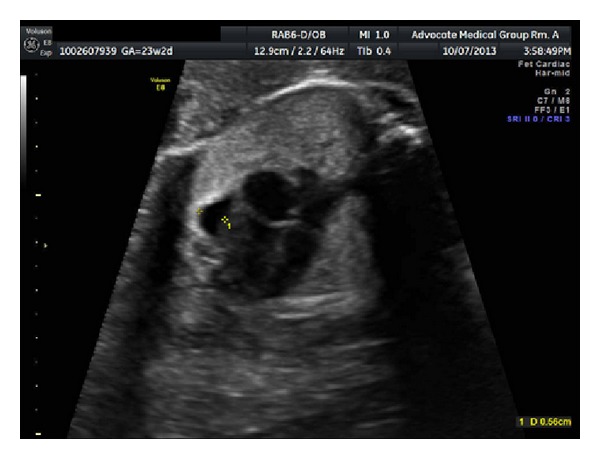
Fetal pericardial effusion.

The patient is a 32-year-old G4P2012 admitted at 23 6/7 weeks of gestation for fetal tachycardia. The fetal heart rate was noted to be persistently between 180 and 190 beats per minute, which is shown in Figure 1. Fetal ECHO revealed a structurally normal heart, with an isolated pericardial effusion which is demonstrated in Figure 2. The patient's past medical history was significant for Graves' disease for which she underwent radioactive iodine ablation 2 years earlier. She became hypothyroid soon thereafter and has been maintained on thyroid replacement. Her current dose is 150 mcg daily. She had two prior full term vaginal deliveries without complication and one first trimester elective abortion. Her past surgical history was significant for a laparoscopic appendectomy. She denied tobacco, alcohol, or illicit drug use. On arrival to labor and delivery, the fetal tachycardia was again noted. Laboratory studies revealed a normal metabolic and thyroid profile. Stimulating thyroid antibodies were drawn but not yet available. The patient had a normal EKG. Because of the persistence of the fetal tachycardia and the pericardial effusion, the decision was made to treat the fetal tachycardia with maternally administered digoxin. Although there was suspicion that the tachycardia may be secondary to thyroid stimulating immunoglobulins (TSIs), the decision was made to start with our usual first-line drug for SVT in the absence of confirmatory results. The patient was loaded with IV digoxin and subsequently placed on an oral maintenance dose of 0.375 mg daily. She was discharged home with close follow-up.

Despite having a maternal digoxin level as high as 2.5 ng/mL, the tachycardia persisted. Over the following week, she complained of increasing nausea. A maternal EKG showed nonspecific changes. The thyroid stimulating antibodies returned significantly elevated at 195% of basal activity. The digoxin was discontinued, and Sotalol 80 mg PO bid was begun. There was no significant improvement over the following few days with the fetal heart rate between 170 and 190 bpm. The Sotalol was increased to 120 mg bid. A few days later the patient complained of decreased fetal movement. A maternal EKG showed a HR of 62. The decision was then made to begin maternal PTU 100 mg three times a day for presumed fetal hyperthyroidism secondary to the transplacental crossing of maternal thyroid stimulating immunoglobulins. Within 48 hours, the fetus had a normal sinus rhythm of 150 bpm. The Sotalol was decreased to 80 mg bid and discontinued the following visit when the FHR was noted to be 140 bpm.

The pericardial effusion resolved over the next few weeks, and the fetal heart rate remained normal for the remainder of the pregnancy. She was maintained on the same dose of PTU, and her thyroid function testing remained normal.

An induction of labor was undertaken at 37 weeks of gestation for presumed fetal hyperthyroidism, which resulted in a vaginal delivery of a live born female infant, with Apgar scores of 9 and 9 after 1 and 5 minutes. The neonate appeared well and in sinus rhythm of 164 bpm. Initial thyroid labs revealed a suppressed TSH of 0.013, a normal free T4 of 1.4 ng/dL, and an elevated free T3 of 5.1 pg/mL. Although the neonate appeared clinically stable, thyroid function tests redrawn at 2 days of age were markedly abnormal, with a TSH of 0.008, free T4 > 8, free T3 > 20, and thyroid stimulating IG 372. By days 4-5 of life she was noted to be tachycardic, jittery, and with loose stools. Methimazole was started at 0.35 mcg every 8 hours. Propranolol 0.5 mg/kg/dose three times a day while being in the hospital for a HR of 180–200, which improved to a baseline of 150 bpm. The neonate was discharged home in stable condition at 1 week of age, with close follow-up. The methimazole was gradually lowered over the following few weeks based on thyroid function testing every 7–10 days. The medication was discontinued at 6 weeks of age.

## 3. Comment

Approximately 1 to 5 percent of mothers with hyperthyroidism caused by Graves' disease have fetuses or neonates with hyperthyroidism. Thyroid stimulating immunoglobulins cross the placental barrier and in high titers can stimulate the fetal thyroid gland, which may result in fetal hyperthyroidism [4]. The fetal thyroid gland hypertrophies and thyrotoxicosis can cause fetal tachycardia, goiter, oligohydramnios, intrauterine growth retardation, and accelerated bone maturation. Cardiac failure and hydrops may also occur with severe disease and can have deleterious effects on neural development. Most neonatal Graves' disease occurs in the setting of active Graves' hyperthyroidism in the mother. However, the disorder also can occur in infants of women with a history of Graves' hyperthyroidism treated with thyroidectomy or radioactive iodine in the past [5]. After a woman with Graves' disease undergoes one of these treatments, the risk of having an infant affected by neonatal Graves' disease falls over time, in conjunction with decreases in immunoglobulin levels. The risk of neonatal Graves' disease is generally low five years after radioactive iodine, but some mothers still have persistent elevation and will deliver babies with neonatal Graves' disease [6]. It is worth emphasizing that, in a woman with Graves' disease who has had surgery or ablation and still has elevated TSIs, the risk of fetal/neonatal Graves' disease is higher than in a woman with elevated TSIs who is taking thionamides, as the fetus is exposed only to TSIs and not to treatment.

With respect to thionamide choice in pregnancy, most clinicians now try to avoid PTU in favor of methimazole after the first trimester due to the risk of maternal agranulocytosis. Furthermore, methimazole crosses the placenta more efficiently. Measurement of maternal serum thyroid stimulating antibodies is warranted in women with active Graves' hyperthyroidism and in women with a history of Graves' disease. Serum thyroid immunoglobulin levels greater than two to three times the upper limit of normal put the fetus at risk for hyperthyroidism [7]. Therefore, fetal heart rate monitoring and serial ultrasounds to check for fetal goiter and growth should be performed when TSIs are elevated [8].

Measuring the fetal concentration of thyroid hormones when a goiter is noted is useful, as this finding can be associated with both hypothyroidism and hyperthyroidism. However, fetal tachycardia in the setting of elevated thyroid stimulating immunoglobulins has not to our knowledge been reported in association with a different etiology. Given the risks of invasive testing and the remote possibility of a different etiology, we did not pursue direct fetal thyroid testing.

Neonatal hyperthyroidism may occur within 24 to 72 hours after delivery in cases of high maternal thyroid stimulating antibody titers, as the antithyroid drug concentrations decrease, while the maternally derived antibodies persist. This is what occurred in our case. Neonatal hyperthyroidism is usually a transient condition, lasting between 3 and 12 weeks as the maternal antibody clears from the infant's circulation. Treatment of the neonate with antithyroid drugs is indicated until resolution of the hyperthyroidism.

In conclusion, this is a relatively uncommon case of fetal and neonatal Graves' disease. It serves as an excellent reminder to clinicians to screen women for TSIs in early pregnancy who have either current hyperthyroid disease or hypothyroid disease secondary to ablation or surgery for Graves' disease. Clinicians should remain vigilant for fetal Graves' disease with fetal heart rate assessment and ultrasound for goiter and growth. The pediatrician caring for the neonate needs to be informed, as neonatal Graves' disease affects 2–5% of neonates born to mothers with a history of Graves' disease due to the transplacental transfer of TSIs.
